# Supplementary material for: Chemotherapy-induced cachexia and model-informed dosing to preserve lean mass in cancer treatment
Source: PLoS Comput Biol. 2022 Mar 21;18(3):e1009505. doi: 10.1371/journal.pcbi.1009505 (PMC8989307; doi:10.1371/journal.pcbi.1009505)
Supplement: S1 Appendix — Sample code is included here to numerically simulate the described mathematical model with τ-day average exposure function and all referenced model parameters. (PDF) [file pcbi.1009505.s001.pdf]

S1 Appendix:
Julia code to simulate mathematical model

Below is sample code to numerically simulate the described mathematical model with the τ-day average exposure function and all referenced model parameters.

```
# Load required packages
using DifferentialEquations, Plots
using QuadGK
using ParameterizedFunctions

# Define function to solve only Chemotherapy PK
function Chemo(du,u,p,t)
  K21,V1,V2,K12,K10 = p
  du[1] = K21*V2/V1*u[2] - K12*u[1] - K10*u[1]
  du[2] = K12*V1/V2*u[1] - K21*u[2]
end

# Define function to build y-tau function given a dose, schedule, and tau value
function buildy(d, tau, doseTimes, tspan) # micro-g/kg dose

    K10 = 151.2; #1/days
    K12 = 5.62 # 1/days
    K21 = 2.31 # 1/days
    V1 = 0.71e3 # ml
    V2 = 0.1e3 # ml
    p = [K21, V1, V2, K12, K10]
    u0 = [d/V1; 0.0]

    ChemSys = ODEProblem(Chemo, u0, tspan, p)

    condition(u,t,integrator) = t in doseTimes
    affect!(integrator) = integrator.u[1] +=  d/V1
    cb = DiscreteCallback(condition,affect!)

    Csol = solve(ChemSys, callback=cb, tstops=doseTimes,
                 reltol=1e-6, abstol=1e-6);

    function intTimes(t)
        starttime = max(t-tau,0)
        [starttime,
        doseTimes[(doseTimes .>= starttime) .& (doseTimes .<=t)]..., t]
    end

    y(t) = quadgk(t -> Csol(t, idxs=2),
             intTimes(t)...; rtol=1e-6, order=7)[1]/tau

    return Csol, y

end

# Define the model parameters
MuscleTumourParameters = NamedTuple{(:p₀, :p₁, :ν₀, :ν₁, :d₀, :m,
                         :k_21, :k_12, :k_10, :V_1, :V_2, :μ, :μ_1,
             :η, :κ),NTuple{15,Float64}}
muscleparams = (p₀=0.479, p₁=0.133, ν₀=0.087, ν₁=5.591, d₀=0.05, m=1000.0,
                k_21=2.31, k_12=5.62, k_10=151.2, V_1=0.71e3, V_2=0.1e3,
                μ=0.446, μ_1 = 116.0, η = 20.0, κ = 0.13)


"""
Build a Cachexia Muscle and Tumour Model from a set of muscle and tumour
parameters and a mean exposure protocol, which is a callable taking 4 real
parameters and outputting 1
```

```julia
"""
function cachexiamodel(mtp::MuscleTumourParameters, y)
    P0, P1, nu0, nu1, D0, M, K21, K12, K10, V1, V2, μ, μ_1, η, κ = values(mtp)
    """
    define cachexia ODE model to be used in ODEProblem with parameters p
    to be fit by optimization p = [R_D],
    u = (stem_volume, muscle_volume, concentration_1, concentration_2,
         tumour_volume)
    """
    function innercachexiamodel(du, u, p, t)
        # Stem Cells
        du[1] = (1 - (y(t)/p[1])^3)*(2*(P0 + P1/(1+u[2]/M))-1)*
                 (nu0 + nu1/(1+u[2]/M))*u[1]
        # Muscle Cells
        du[2] = (1 - (y(t)/p[1])^3)*2*(1-(P0 + P1/(1+u[2]/M)))*
                 (nu0 + nu1/(1+u[2]/M))*u[1] - D0*u[2]
        # Concentration C1
        du[3] = K21*V2/V1*u[4] - K12*u[3] - K10*u[3]
        # Concentration C2
        du[4] = K12*V1/V2*u[3] - K21*u[4]
        # Tumour Volume
        du[5] =  μ*u[5]*( 1 + (μ/μ_1*u[5])^(η) )^(-1/η) - κ*u[4]*u[5]
    end
    return innercachexiamodel
end


# Define Parameters and Model Problem
ξ = 0.002 # g/mm^3 volume-mass conversion factor
Rd = 6.8 # μg/kg/ml fitted parameter value
p = [Rd] # parameters to be fit by optimization over all dose curves
tau = 8 # number of days to average C2 concentration over

# Define Initial Conditions
# Data from Makino et al, mice 6-wk old male CDF1 ave mass 25g
S0 = 267.5 # 6-wk old mice according to stem ratio
M0 = 4732.5 # 6-wk old mice according to stem ratio
V1 = 0.71e3 # ml from Chemotherapy model
T0 = 10 # mm^3 initial Tumour Volume

# Define daily standard dose
dose = 24e3 # μg/kg
dosetimes = [1:1:27;] # Daily schedule dosing on days 0 to 29 (30 days on)
# Alternate standard dose
#dose = 35e3 # μg/kg
#dosetimes = [1:1:4;7:1:11;14:1:18;21:1:25;] # 5-on, 2-off schedule

u0 = [S0; M0; dose/V1; 0; T0] # initial conditions for model
tspan = (0.0, 60.0) # time span to solve model over

# build y-tau average exposure function
Csol, y = buildy(dose, tau, dosetimes, tspan)

# Plot chemotherapy dynamics
P1 = plot(Csol,vars=(1),label="C1", lw=4, plotdensity = 600000)
plot!(Csol,vars=(2),label="C2", title ="Drug Concentration", xlabel="",
    ylabel="μg/kg/ml", lw =4, plotdensity = 600000)
# Plot average exposure function
P2 = plot(t -> y(t), 0:60, lw = 4, title = "Average Daily Exposure",
    label="y(t,"*string(tau)*")")
l = @layout [a{0.7w} b]
plot(P1, P2; layout = l, size = (750,200))

# Simulate Control  -  no chemotherapy
dose = 0.0 # μg/kg
u0 = [S0; M0; dose/V1; 0; T0]
dosetimes = []
```

```
y = buildy(dose, tau, dosetimes, tspan)[2]
DEsys = ODEProblem(cachexiamodel(muscleparams,y), u0, tspan, p)
condition(u,t,integrator) = t in dosetimes
affect!(integrator) = integrator.u[3] +=  dose/V1
cb = DiscreteCallback(condition,affect!)
ctlSoln = solve(DEsys, callback=cb, tstops=dosetimes, reltol=1e-6, abstol=1e-6);

# Compute Lean Mass
LeanMass = [(t,ξ*(u[1]+u[2])) for (u,t) in tuples(ctlSoln)]

# Plot Lean mass under control situation
P1 = plot(LeanMass, label = "Control", color=:black,
     legend=:outerright, lw=4,
     title = "(a)  Lean Mass Under Chemotherapy",
     ylabel="Lean Mass (g)", xlabel="Time (days)",
     xlims=(0,60), ylims = (7,13))
# Plot tumour volume under control situation
P2 = plot(ctlSoln, vars=(5), lw=4, label="Control", legend=:outerright,
     color=:black, linestyle = :solid, yaxis=:log, ylims=(0.1, 1e4),
     title="(ii)  Tumour Volume Under Chemotherapy", ylabel="Volume (mm^3)",
     xlabel="Time (days)")

# Plot daily standard schedule 24 mg/kg/dose
dose = 24e3 # μg/kg
u0 = [S0; M0; dose/V1; 0; T0]
dosetimes = [1:1:27;] # daily dosing over 28 days including IC

y = buildy(dose, tau, dosetimes, tspan)[2]
DEsys = ODEProblem(cachexiamodel(muscleparams,y), u0, tspan, p)
condition(u,t,integrator) = t in dosetimes
affect!(integrator) = integrator.u[3] +=  dose/V1
cb = DiscreteCallback(condition,affect!)
soln = solve(DEsys, callback=cb, tstops=dosetimes, reltol=1e-6, abstol=1e-6);

# Compute lean mass
LeanMass = [(t,ξ*(u[1]+u[2])) for (u,t) in tuples(soln)]

# Plot lean mass under treatment
plot!(P1, LeanMass, lw=4, label="24 Daily", color=:blue, linestyle=:dash)
# plot tumour volume under treatment
plot!(P2, soln, vars=(5), lw=4, label="24 Daily",
     color=:blue, linestyle=:dash, legend=:false)

l = @layout [a{0.6w} b]
plot(P1, P2; layout = l, size = (750,300))
```
