# Supplementary material for: Chemotherapy-induced cachexia and model-informed dosing to preserve lean mass in cancer treatment
Source: PLoS Comput Biol. 2022 Mar 21;18(3):e1009505. doi: 10.1371/journal.pcbi.1009505 (PMC8989307; doi:10.1371/journal.pcbi.1009505)
Supplement: S2 Appendix — The local sensitivity analysis of the lean mass model is presented here after a full schedule of 35 mg/kg daily dosing. (PDF) [file pcbi.1009505.s002.pdf]

S2 Appendix:
Local sensitivity analysis of the model

Here we present the local sensitivity analysis of the lean mass model on day 28 after a full schedule of 35 mg/kg daily dosing on days $0, \ldots, 27$. Each parameter in Eqs (1), (2), (7) and (8) was considered and perturbed by factor $\epsilon = 10^{-5}$. The sensitivity coefficients are presented in Fig 1. We exclude the tumour growth parameters since our focus is on the response of lean mass, and there is no coupling between the tumour equation and the lean mass equations in this model.

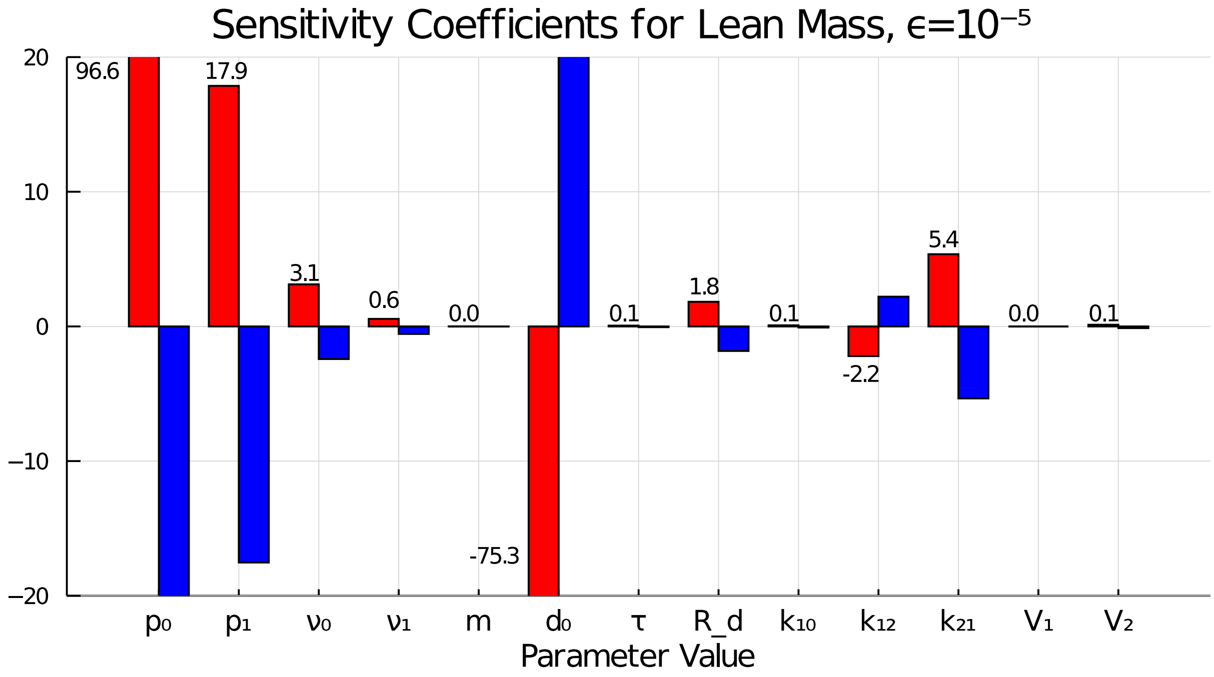

Figure 1: Parameter sensitivity analysis for model parameters that affect the lean mass. Calculation is performed on day 28 following a 35 mg/kg daily chemotherapy schedule on days $0 - 27$. A value of $\epsilon = 10^{-5}$ is used to calculate the sensitivity coefficents.
