# Supplementary material for: Chemotherapy-induced cachexia and model-informed dosing to preserve lean mass in cancer treatment
Source: PLoS Comput Biol. 2022 Mar 21;18(3):e1009505. doi: 10.1371/journal.pcbi.1009505 (PMC8989307; doi:10.1371/journal.pcbi.1009505)
Supplement: S3 Appendix — A comparison is presented here between our daily and (5-on, 2-off) schedules and maximum tolerated dosing schedules delivering high doses weekly, biweekly, or monthly. (PDF) [file pcbi.1009505.s003.pdf]

S3 Appendix:
Maximum tolerated dosing schedules

We briefly compare the daily and (5-on, 2-off) schedules to maximum tolerated dosing on weekly, biweekly, or monthly schedules. The comparison is shown in Fig 1. We assume a bolus injection equivalent to our weekly doses of 168 mg/kg/week from the 24 mg/kg daily schedule, and 175 mg/kg/week from the 35 mg/kg (5-on, 2-off) schedule. Each of these weekly dosages is applied either weekly, biweekly (336 mg/kg/2 weeks or 350 mg/kg/2 weeks), or monthly (672 mg/kg/month or 700 mg/kg/month). An additional monthly dose of 510 mg/kg/month was added for comparison as this monthly dose achieved an equivalent tumour volume AUC as the daily schedule. Note that with biweekly or monthly dosing, the effect of the τ-day averaging is lost in muscle response, since the time interval between doses is larger than $\tau = 8$. The larger doses are predicted to induce greater tumour response with greatly increased lean mass loss. In terms of therapeutic efficacy, the bi-weekly regimes perform best, but cause $30 - 40\%$ lean mass loss during treatment.

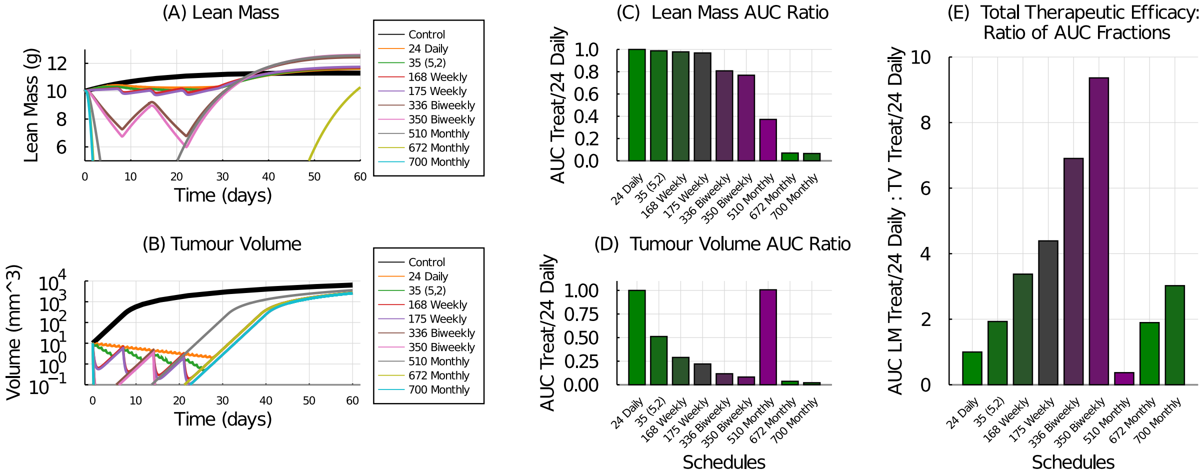

Figure 1: Model prediction for monthly, biweekly, and weekly schedules delivering equivalent doses to the daily (168 mg/kg/week) or (5-on, 2-off) (175 mg/kg/week) schedules. Lean mass (A) and tumour (B) response to treatment schedules. (C) The AUC ratio for lean mass compares the lean mass response from the tested schedule to that of the 24 mg/kg daily schedule. (D) The AUC ratio for tumour volume compares the tumour volume response from the tested schedule to that of the 24 mg/kg daily schedule. (E) The total therapeutic efficacy is the ratio of the lean mass AUC ratio to the tumour volume AUC ratio.
